# The prevalence of corneal abnormalities in first‐degree relatives of patients with keratoconus: a prospective case‐control study

**DOI:** 10.1111/opo.12706

**Published:** 2020-07-24

**Authors:** Einat Shneor, Joseph Frucht‐Pery, Edna Granit, Ariela Gordon‐Shaag

**Affiliations:** ^1^ Department of Optometry and Vision Science Hadassah Academic College Jerusalem Israel

**Keywords:** genetics, keratoconus

## Abstract

**Purpose:**

Although there is a high prevalence of keratoconus in the Middle East including Israel, limited data is available describing first‐degree relatives of patients with sporadic keratoconus (KC) using Scheimpflug imaging. The purpose of this study is to accurately phenotype first‐degree relatives of patients with sporadic KC in Israel using corneal tomography, which may help determine the genetic aetiology of KC.

**Methods:**

First‐degree relatives (*N* = 56) of 16 KC probands participated in this prospective case‐control study. Healthy controls (*N* = 96) were from a previous study. Autorefraction, visual acuity, slit lamp biomicroscopy, retinoscopy, subjective refraction and Scheimpflug imaging (CSO Sirius Topographer) of keratoconus patients and their first‐degree relatives were evaluated. The worse eye was used for KC and KC suspects. The main outcome measure was prevalence of abnormal corneal topography and tomography parameters, which was compared between first‐degree relatives vs controls. *p* values < 0.05 were considered significant.

**Results:**

KC (*N* = 2) or KC suspect (*N* = 8) was diagnosed in 18% (95% CI 8‐28%) of the first‐degree relatives. At least one abnormal corneal parameter was evident in 34% of first‐degree relatives, while this was significantly lower for controls (14%, χ^2^_(1,_ *_N _*_= 152)_ = 8.8, *p* = 0.01). Qualitative analysis showed KC first‐degree relatives had significantly more abnormal anterior corneal topography patterns than controls (34% vs 17%, χ^2^_(1,_ *_N_*_ = 152)_ = 5.9, *p* = 0.02). For first‐degree relatives, sex was not a factor influencing prevalence of corneal abnormalities (18% for both men and women, χ^2^_(1,_ *_N_*_ = 56)_ = 0.0, *p* = 1.0). A significant correlation was found for first‐degree relatives between age and most corneal parameters, while this was not evident for the control group.

**Conclusions and Relevance:**

Eye care practitioners should consider first‐degree relatives of patients with KC at moderate risk for the disease and/or corneal abnormalities.

## Introduction

Keratoconus (KC) is a bilateral progressive corneal disease that usually starts at puberty and is characterised by vision deterioration, irregular astigmatism and corneal thinning^1^ and may lead to a protrusion in the cornea, high myopia and irregular astigmatism.^2^ As a progressive disease, it usually becomes apparent in the second decade of life,^2^, ^3^ but can develop earlier,^4^, ^5^ and tends to stabilise by the fourth decade.^6^ Early detection of keratoconus is crucial since collagen crosslinking treatment may stop the progression of the disease^7^ and as a counterindication for refractive surgery.^8^

The diagnosis of KC is largely based on clinical, topographical and tomographical findings.^9^, ^10^ Advanced tomographic instruments such as rotating Scheimpflug cameras combined with Placido disk or slit‐scanning devices can provide assessment of topographic and tomographic properties of the anterior and posterior cornea, including pachymetric, corneal power and elevation measurements.^11^, ^12^ There is a global consensus among cornea experts that corneal tomography is the most sensitive method for early diagnosis, monitoring progression, and treatment of KC.^10^

The prevalence of KC in the general population is varied, but has been shown to be about 2‐3% in the Middle East, in Israel,^13^, ^14^ Palestine,^15^ Lebanon,^16^ Saudi Arabi^17^ and Iran.^18^, ^19^, ^20^

The aetiology of KC is still unclear, and it is assumed that KC has both an environmental and a genetic basis.^21^ Environmental factors include the use of contact lenses,^22^ eye rubbing, ^21^, ^23^, ^24^ allergy, asthma and eczema.^21^ Evidence for a genetic aetiology comes from a higher concordance rate of KC in monozygotic twins^25^ and strong association of parental consanguinity/endogamy with the disease.^23^, ^26^ Furthermore, family history of KC has been shown as a risk factor in many studies.^27^, ^28^, ^29^, ^30^, ^31^

Despite clear indications of a genetic aetiology, identification of specific KC genes remains elusive. Many genes have been reported to be linked or associated with KC, but the evidence for a pathogenic role for most remains limited.^32^ Furthermore, if the involvement of the reported genes in KC development can be proven, they only account for a limited number of patients.^32^ One possible explanations is that KC is a disease continuum: at one end of the spectrum are families in which KC is a monogenetic disease with high penetrance and at the other are populations in which the disease is caused by environmental factors in combination with a large number of small effect genetic risk factors and can thus be considered as a complex disease.^32^

To differentiate the place on the KC spectrum, each family and population should be analysed to identify specific subtle corneal phenotypes using videokeratography and tomography, including both anterior and posterior elevation and pachymetric data.^33^ This process of accurate phenotyping of the cornea has been carried out in a limited number of populations using videokeratography^34^, ^35^, ^36^, ^37^ or tomography^38^, ^39^, ^40^, ^41^. While these papers show a high prevalence of KC or corneal abnormalities in relatives of patients with KC they are limited in scope: only one looked at first‐degree relatives in families with sporadic KC (i.e. only one family member was diagnosed with KC at the time of recruitment),^39^ while the rest either analysed extended families,^41^ pedigrees with familial keratoconus^40^ or limit the scope to paediatric family members.^38^ Furthermore, there are no papers describing the corneal phenotypes of KC family members in Israel. The purpose of this study is to accurately phenotype first‐degree relatives of patients with sporadic KC in Israel using corneal tomography. We hypothesise that first‐degree relatives of patients with sporadic KC will exhibit a higher prevalence of corneal abnormalities when compared to control patients. The results of this study will help determine the genetic aetiology of KC in Israel.

## Methods

### Subjects

This study was approved by the Hadassah Academic College (HAC) Ethics Committee and followed the tenets of the declaration of Helsinki. Three study groups have been included: (1) KC patients (KC proband) at the HAC Eye Clinic who all receive counselling recommending bringing in first‐degree relatives (i.e., parents, siblings and/or children) for a full exam to determine their KC status. (2) First‐degree relatives of the KC probands who complied with counselling. (3) Normal controls (right eyes) were used from a database from a previous study.^42^ Six subjects were removed since the left eye did not have enough data to classify as being normal.

Subjects for groups one and two were recruited from March 2015 to January 2020. All families, aside from one, were sporadic at the time of recruitment. Demographic and clinical parameters were described for all groups; however, to answer the aims of this study, only first‐degree relatives and controls were compared.

### Exclusion criteria

KC probands were excluded if they had any systemic or ocular conditions positively or negatively associated with KC.^30^ Subjects who had corneal graft surgery for KC were categorised as KC based on previous medical records. For all subjects, hard contact lens wear was stopped the night prior examination and soft contact lens wear was stopped half an hour before. However, some patients with KC cannot cope without contact lenses. In these situations, contact lens wear was not stopped and KC was diagnosed based on previous medical records as well as imaging. Subjects with known epileptic history were also excluded, as well as children who were deemed too young to cooperate with the entire exam.

All examinations took place at the HAC eye clinic. The methods were orally explained to the participants and they signed a statement of informed consent prior to their participation (for children‐ assent and guardian consent).

### Procedures

The corneal phenotype was characterised in first‐degree relatives of KC probands by a complete ocular exam. This included autorefraction (L80+, http://www.skymed.co.il/l80‐visionix.html), autokeratometry, corneal topgraphy/tomography (Sirius, https://www.csoitalia.it/en/prodotto/info/47‐sirius#), visual acuity (Snellen) and subjective refraction. Slit lamp biomicroscopy and retinoscopy were performed to evaluate clinical signs of KC. Each exam was performed by a licensed optometrist and the diagnosis confirmed by an ophthalmologist with a specialty in cornea (JFP).

Diagnosis criteria used for first‐degree relatives and controls: A diagnosis of KC was based on abnormal topography or tomography and at least one of the following signs:^2^ stromal thinning, Munson’s sign, Fleischer’s ring, or Vogt’s striae, observed by slit‐lamp examination or scissor reflex observed by retinoscope. A subject with KC in at least one eye was defined as having the disease. The criteria for KC suspects was abnormal topography and at least one corneal tomography defect (listed below) but without clinical signs. For KC and KC suspect, the worse eye was used (based on curvature and central corneal thickness (CCT)) for analyses. For example, if a subject has one eye with KC and the other was KC suspect, he was classified as KC and only the data of that eye was used for analysis. However, in some cases, the worse eye had undergone a cornea transplant or was not able to undergo imaging, in which case the better eye was included in the analysis. For first‐degree relatives, the worse eye was used (based on curvature and CCT) unless no clinically significant differences were noted in tomography between eyes and then the right eye was used.

Normal and abnormal Sirius corneal tomography parameters were based on the only study that used the Sirius to analyse a large cohort of KC, KC suspect, post corneal surgery and normal eyes.^43^ The Sirius combines a Placido disc topographer with a rotating Scheimpflug camera. Anterior corneal topography is based primarily on the Placido disc, while thickness, elevation, and the posterior corneal curvature are derived from the Scheimpflug camera.^44^ This paper described the following parameters for all groups: thinnest corneal thickness (TCT), symmetry index front (SIf), symmetry index back (SIb), Baiocchi Calossi Versaci front (BCVf), Baiocchi Calossi Versaci front (BCVb), root mean square front (RMSf) and root mean square back (RMSb). A parameter was considered abnormal if it was outside the 95^th^ or 5^th^ percentile of the 1269 normal eyes analysed.^43^ In addition, clinical parameters that are typically used to describe KC subjects were analysed such as posterior and anterior keratometry and corneal apex and CCT.

Qualitative analysis of anterior cornea patterns was performed in order to classify a topography pattern as normal or abnormal, based on Rasheed *et al*.^45^ This type of analysis will allowed the comparison of the corneal topography patterns of subjects in this study to previous research.^35^, ^40^ This was performed by two separate masked observers (AGS and ES) who classified the images according to the technique described in Bogan, *et al*.^46^ A pattern was considered skewed if it had more than 30 degrees between the axes bisecting the lobes of the superior and inferior bowtie,^2^ as measured with a compass. Observers agreed in 86.4% of eyes and a third observer (AB) was asked to measure the images in cases where disagreement arose. Topographical patterns were classified as normal if they were round, oval, symmetric bowtie, asymmetric bowtie with superior steepening, asymmetric bowtie with inferior steepening or superior steepening. Topographic patterns were classified as abnormal if they were symmetric bowtie (SB) with skewed radial axes (SRAX), asymmetric bowtie (AB) with skewed radial axes, inferior steepening or irregular. Only bowtie patterns with skewed radial axes were considered abnormal since the line of sight and the measurement axis of the videokeratoscope are not the same. Thus, a change in the reference axis can create different axial curvature maps from the same shape turning a SB into AB.^12^

### Statistical analysis

#### Sample size calculation

The main outcome measure is comparison of the prevalence of first‐degree relatives with abnormal corneas with the prevalence of controls with abnormal corneas: The minimum sample size was based on an assumed average prevalence of KC in first‐degree relatives of 20% (based on questionnaires in previous research in Israel)^23^, ^47^ and a prevalence of KC in the general population of 3%^13^, ^14^ with a 95% confidence level and 80% power. This calculation^48^ resulted in a minimum required sample size of 52 subjects in each group to get a statistically significant difference.

Descriptive statistics were used to calculate mean, standard deviation (S.D.) and 95% confidence intervals (CI). Chi‐square was used to compare the prevalence of KC/KC suspect status, different corneal abnormalities (both qualitative Sirius indices and the quantitative analysis of corneal topography patterns described in the previous section) and gender between first‐degree relatives and controls. For prevalence comparisons in categories for which *N* was less than five, Fisher’s exact test was used. First‐degree relatives and controls were compared for continuous variables using the t‐tests if the normality assumption was satisfied. Normality was checked with Anderson‐Darling and Mann‐Whitney U test was used when data did not meet normality criteria. Chi‐square was used to compare the prevalence of corneal abnormalities between genders within the cohort of first‐degree relatives.

Pearson correlation was calculated to test the correlation between age and corneal parameters for both KC first‐degree relatives and healthy controls.

Statistics were calculated using SPSS version 25 (https://www.ibm.com/uk‐en/analytics/spss‐statistics‐software). *p* ≤ 0.05 was considered statistically significant.

## Results

Sixteen KC probands brought 56 first‐degree relatives for examination. Thirteen of the KC probands had bilateral KC and the remaining three had unilateral KC. Demographic and clinical parameters of the KC probands are described in *Table* *1*. First‐degree relatives included seven parents (13%), 25 siblings (46%) and 22 (41%) children. On average, 3.3(2.3) first‐degree relatives per family (range 1‐8) participated in this study. Demographic and refractive data of 56 first‐degree relatives and healthy controls are described in *Table* *2*. KC was diagnosed in two first‐degree relatives (prevalence 4%; 95% confidence interval (CI): 1‐9%, one was bilateral). KC suspect was diagnosed in at least one eye of eight first‐degree relatives (prevalence 14%; 95% CI 5‐25%, only one first‐degree relatives was KC suspect in both eyes). Altogether, 18% (95% CI 8–28%) of the first‐degree relatives were KC or KC suspect.

**Table 1:** Demographics and clinical parameters of Keratoconus probands

KC patients	
Number of Participants	*N* = 16
Number of Females (%)	*N* = 6 (38%)
	**Mean (S.D.)**
Age; years	35.1 (14.8)
Sphere; D	−3.6 (3.5)
Cylinder; D	−3.0 (2.2)
VA	6/5 to 6/36+
Anterior K1; mm	7.1 (0.7)
Anterior K2; mm	6.7 (0.7)
Average Anterior K; mm	6.9 (0.7)
Posterior K1; mm	5.6 (1)
Posterior K2; mm	5.1 (0.9)
Average Posterior; mm	5.3 (0.9)
Front Apex Thickness; µm	464.7 (75.2)
Front Apex Curve; mm	5.6 (0.9)
Back Apex Curve; mm	4.0 (0.8)
CCT; µm	457.2 (63.3)
TCT ; µm	438.2 (70.9)
SIf	8.2 (4.7)
SIb	2.1 (1.1)
BCVf	4.4 (3.1)
BCVb	4.7 (2.6)
RMSf; µm	21.3 (14.0)
RMSb; µm	41.8 (25.0)

Abbreviations: BCVb, Baiocchi Calossi Versaci back; BCVf, Baiocchi Calossi Versaci front; CCT, central corneal thickness; K, keratometry reading; KC, keratoconus; RMSb, root mean square back; RMSf, root mean square front; S.D., standard deviation; SIb, symmetry index back; SIf, symmetry index front; TCT, Thinnest corneal thickness; VA, Visual Acuity.

**Table 2:** Description of cohort: first‐degree relatives of KC proband and Controls (from previous study)^42^

	First‐degree relatives	Healthy control	*p*
Number of Participants	*N* = 56	*N* = 96	
Number of Females (%)	*N* = 28 (50%)	*N* = 76 (79%)	χ^2^ = 13.9; <0.0001^†^
Mean Age (S.D.) range; years	21.9 (14.6) 6–63	23.0 (5.0) 18–47	0.59^‡^
Mean Sphere (S.D.) range; D	−2.4 (2.2) −9.0 – 1.0	−1.5 (2.5) −11.0 – 4.3	0.71^‡^
Mean Cylinder (S.D.) range; D	−0.8 (0.7) −3.0 – −0.3	−0.7 (0.5) −2.44 – −0.1	0.08^‡^
VA	6/5+ to 6/30+	Not tested	
Anterior K1 (S.D.); mm	7.7 (0.3)	7.7 (0.2)	0.29^§^
Anterior K2 (S.D.); mm	7.6 (0.3)	7.6 (0.2)	0.50^§^
Average Anterior K (S.D.); mm	7.6 (0.3)	7.7 (0.2)	0.38^§^
Posterior K1 (S.D.); mm	6.6 (0.3)	6.6 (0.2)	0.05^§^
Posterior K2 (S.D.); mm	6.3 (0.3)	6.2 (0.2)	0.06^§^
Average Posterior (S.D.); mm	6.4 (0.3)	6.4 (0.2)	0.04^§^
Front Apex Thickness (S.D.); µm	559.5 (57.5)	580.7 (54.8)	0.02^§^
Front Apex Curve (S.D.); mm	7.3 (0.5)	7.4 (0.3)	0.46^§^
Back Apex Curve (S.D.); mm	5.7 (0.8)	5.8 (0.3)	0.48^§^
CCT (S.D.); µm	533.6 (41.0)	544.9 (32.2)	0.03^‡^
TCT (S.D.); µm	529.6 (43.3)	542.1 (32.2)	0.02^‡^
SIf (S.D.)	0.6 (0.7)	0.2 (0.5)	0.11^§^
SIb (S.D.)	0.2 (0.2)	0.0 (0.1)	0.01^§^
BCVf (S.D.)	0.4 (0.4)	0.2 (0.2)	0.01^§^
BCVb (S.D.)	0.3 (0.3)	0.1 (0.1)	0.03^§^
RMSf (S.D.); µm	4.2 (4.1)	2.4 (1.0)	0.01^§^
RMSb (S.D.); µm	9.6 (6.6)	6.3 (2.0)	<0.0001^§^

*p* value is between family members and healthy controls. Abbreviations: KC, keratoconus; S.D., standard deviation; K, keratometry reading; CCT, central corneal thickness; TCT, Thinnest corneal thickness; SIf, symmetry index front; SIb, symmetry index back; BCVf, Baiocchi Calossi Versaci front; BCVb, Baiocchi Calossi Versaci back; RMSf, root mean square front; RMSb, root mean square back.

^†^ Chi‐square test (1, *N* = 152).

^‡^ T‐test.

^§^ Mann Whitney U test.

A statistically significant difference was found between the mean Sirius corneal parameters of KC first‐degree relatives and healthy controls for the following parameters (*Table* *2* and *Tables* S1a,b): Posterior flat keratometry (K1, mm); Average Posterior K (mm); Front Apex Thickness (µm); TCT, CCT, SIb, and all elevation parameters (BCVf, BCVb, RMSf, RMSb).

**Figure 1:**
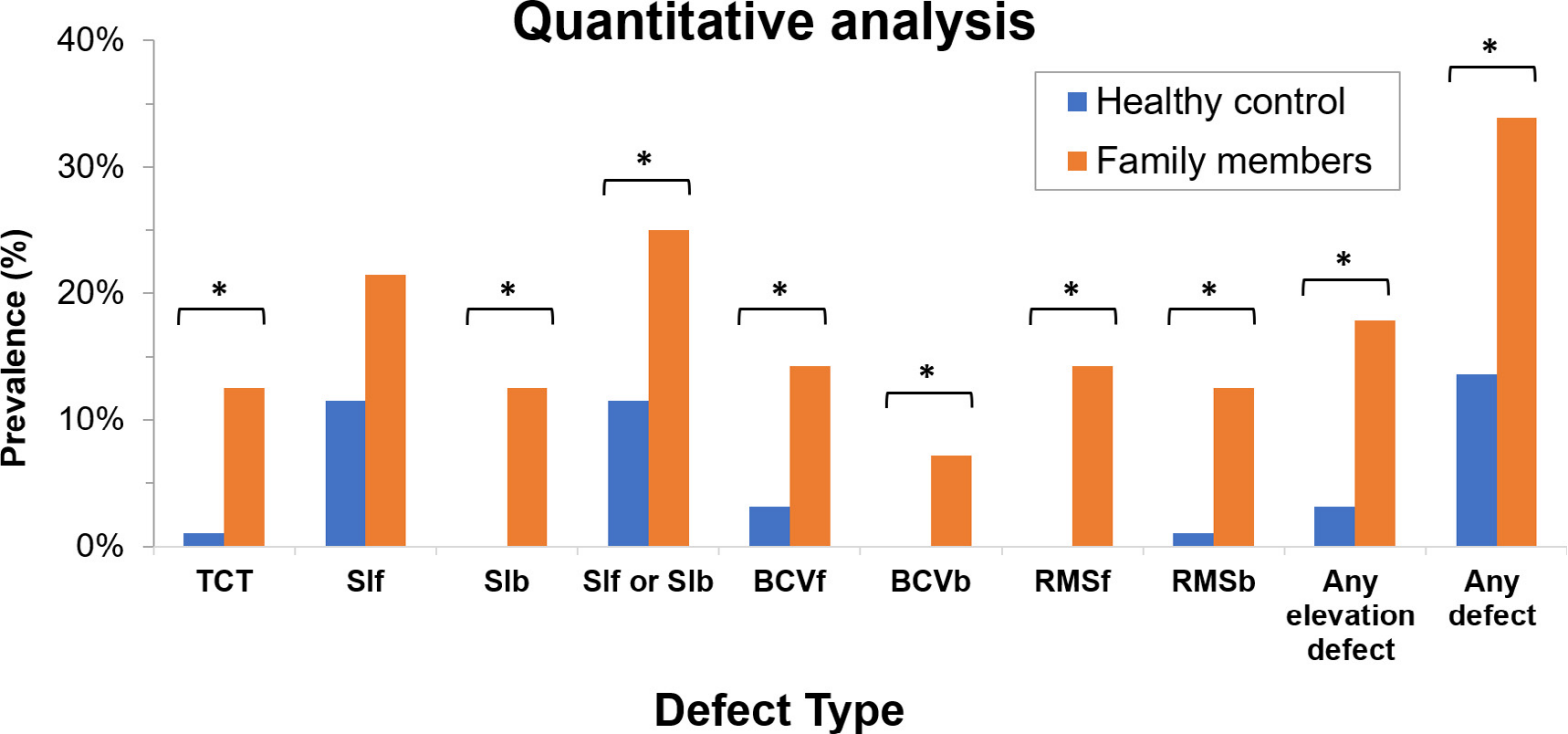
Quantitative analysis of corneal abnormalities. Blue bars represent normal healthy control subjects and orange bars represent First‐degree relatives of keratoconus subjects. Abbreviations: TCT, Thinnest corneal thickness; Sif, symmetry index front; Sib, symmetry index back; RMSf, root mean square front; RMSb, root mean square back; BCVf, Baiocchi Calossi Versaci front; BCVb, Baiocchi Calossi Versaci front. Any elevation defect represents a defect at least one of the following parameters: BCVf, BCVb, RMSf and RMSb. Any defect represents indicates subjects with at least one of the other abnormalities in the figure. Asterisk represents a significant difference between first‐degree relatives and healthy controls (Chi‐square or Fisher’s exact test, see *Table* S2 for details).

The prevalence of different posterior and anterior corneal curvature abnormalities (SIf and SIb) was significantly higher in the KC first‐degree relatives, than in controls (*Figure* *1*, 25% vs 12%, χ^2^ = 4.69, *p* = 0.03). Posterior and anterior elevation defects as shown by BCVf, BCVb, and RMS back were each significantly more prevalent in the KC first‐degree relatives than in controls (*Figure* *1* and *Table* S2). The prevalence of any elevation abnormality was 18% vs 3% for KC first‐degree relatives and controls, respectively (*p* < 0.01). For KC first‐degree relatives, 34% had at least one abnormal corneal parameter, while this was 14% for controls (χ^2^ = 8.8, *p* = 0.01).

The number of Sirius parameter abnormalities in individual eyes in first‐degree relatives and controls was compared (*Table* *3*). First‐degree relatives had significantly more abnormalities per eye than controls (χ^2^ = 15.00; *p* < 0.0001). When abnormalities were present, controls had only one or two per eye while almost half of the first‐degree relatives had three or more abnormalities.

**Table 3:** Abnormalities in individual eyes in controls and first‐degree relatives

	Eyes (*n*)	Normal % (*n*)	1 abnormality, % (*n*)	2 abnormality, % (*n*)	3 abnormality, % (*n*)	4 and more abnormality, % (*n*)
Controls	96	86% (83)	10% (10)	3% (3)	0% (0)	0% (0)
First‐degree relatives	56	66% (37)	14% (8)	5% (3)	4% (2)	11% (6)

**Figure 2:**
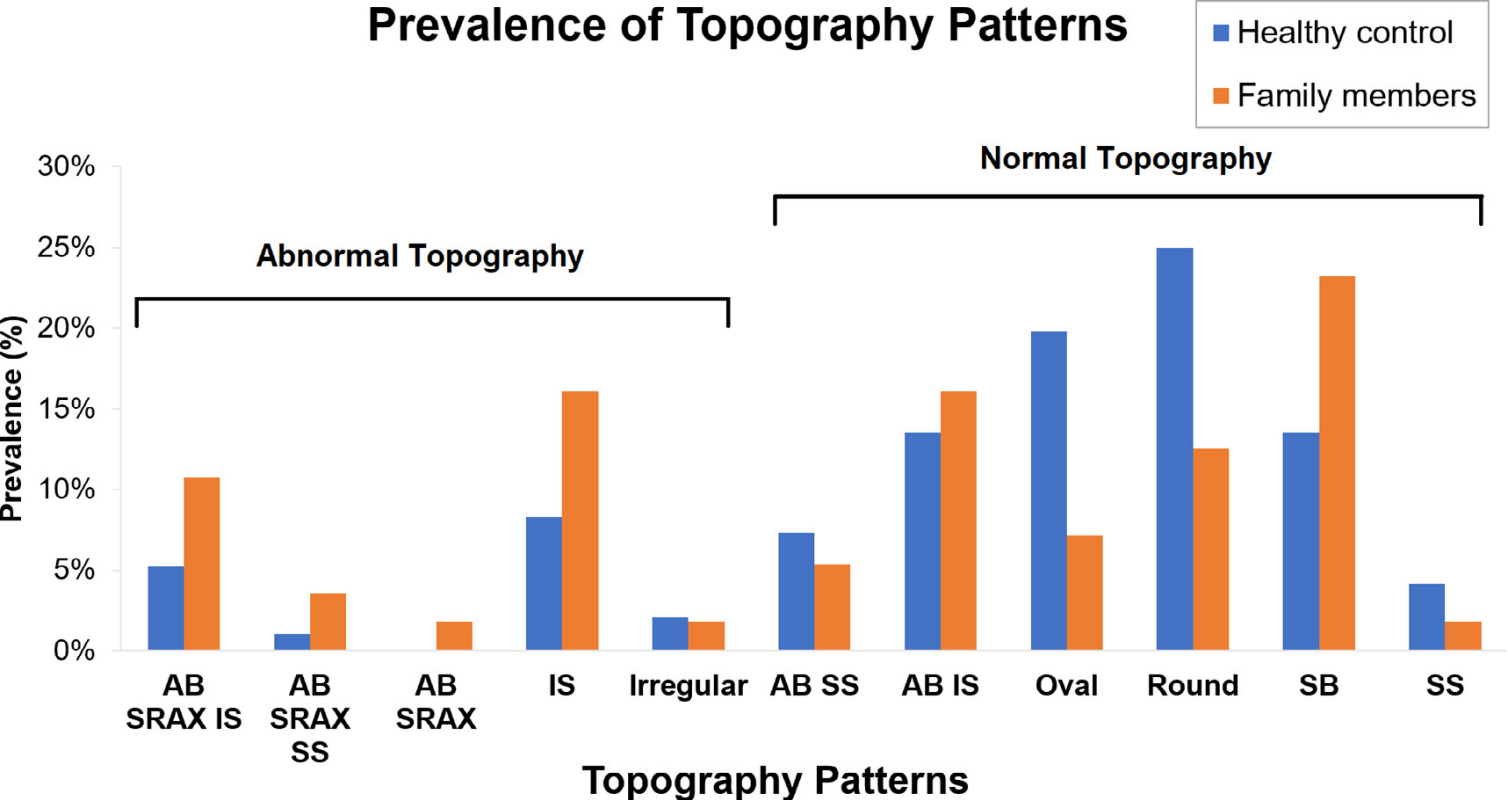
Prevalence of topography patterns in family members and normal controls. Abbreviations: SB, symmetric bowtie; AB, asymmetric bowtie; SRAX, with skewed radial axes; IS, inferior steepening; SS, superior steepening.

Qualitative analysis showed KC family members had significantly more abnormal anterior corneal topography patterns than controls (*Figure* *2* and *Table* S3; 34% vs 17%, χ^2^_(1,_ *_N _*_= 152)_ = 5.91, *p* = 0.02).

To test if abnormal corneal parameters were linked with sex, we calculated the prevalence KC, KC suspect and abnormal corneal parameters for KC first‐degree relatives separately for men and women. Half of the family participants in this study were female. We found that for the KC family members, there was no statistically significant difference between males and females in terms of the prevalence of KC or KC suspect, corneal parameter abnormalities or anterior curvature abnormalities (*Table* *4*).

**Table 4:** Prevalence of abnormalities in keratoconus family members according to sex

Parameter	Female (*N* = 28)	Male (*N* = 28)	Chi square *p* value* (1, *N* = 56)
Prevalence of KC or KC suspect	18%	18%	χ^2^ = 0.00; *p* = 1.00
Any corneal parameter defect	43%	25%	χ^2^ = 1.47; *p* = 0.23
Abnormal Qualitative topography	29%	39%	χ^2^ = 0.72; *p* = 0.40

*p* value is between family members and healthy controls. Abbreviations: KC, keratoconus; χ^2^, Chi‐square test.

Linear regression showed that KC first‐degree relatives exhibited a statistically significant correlation between age and several corneal parameters, while controls for the most part did not (*Table* *5*). For KC first‐degree relatives there was a negative correlation between age and anterior K2, average anterior K, posterior K1, posterior K2, average posterior K, front and back apex curvatures and a positive correlation between age and SIf, BCVf, BCVb, RMSf and RMSb. For the control group, the only significant correlation was between age and BCVb.

**Table 5:** Correlation between age and corneal parameters

	KC first‐degree relatives		Controls	
	*N* = 56		*N* = 96	
	Pearson Correlation	*p* value	Pearson Correlation	*p* value
Anterior K1	−0.25	0.07	0.08	0.42
Anterior K2	−0.29	0.03^†^	0.11	0.30
Average Anterior K	−0.28	0.04^†^	0.10	0.35
Posterior K1	−0.41	0.002^†^	0.08	0.42
Posterior K2	−0.46	<0.0001^†^	0.15	0.14
Average Posterior K	−0.45	0.001^†^	0.12	0.23
Front Apex Thickness	0.06	0.66	−0.20	0.06
Front Apex Curve	−0.44	0.001^†^	0.17	0.09
Back Apex Curve	−0.37	0.01^†^	0.16	0.11
CCT	−0.04	0.76	−0.11	0.29
TCT	−0.07	0.61	−0.10	0.35
SIf	0.34	0.01^†^	0.09	0.38
SIb	0.25	0.07	0.08	0.46
BCVf	0.29	0.03^†^	0.00	1.00
BCVb	0.29	0.03^†^	0.21	0.04^†^
RMSf	0.33	0.01^†^	−0.07	0.51
RMSb	0.34	0.01^†^	0.02	0.82

Significant *p* value is when the correlation is significant between age and the measured parameter.

Abbreviations: BCVb, Baiocchi Calossi Versaci back; BCVf, Baiocchi Calossi Versaci front; CCT, central corneal thickness; K, keratometry reading; KC, keratoconus; RMSb, root mean square back; RMSf, root mean square front; S.D., standard deviation; SIb, symmetry index back; SIf, symmetry index front; TCT, Thinnest corneal thickness.

^†^ Sig. (2‐tailed).

## Discussion

This study provided accurate and specific phenotypic description of the corneas of first‐degree relatives of patients with keratoconus (KC) using corneal tomography. The results of this study may help determine the genetic aetiology of KC in Israel. The prevalence of manifest keratoconus in this cohort (4%) was similar to that found in the general population in Israel (2.3‐3.3%).^13^, ^14^ In contrast, the prevalence of KC suspect in first‐degree relatives (14%) was much higher than previously reported for this population (0.5%).^13^ Thirty‐two percent of KC relatives had abnormal anterior topography and a third had at least one defect in corneal tomographic parameters measured with the Sirius. While the prevalence of these issues has not been addressed in a large population‐based study in Israel, it is significantly higher than in a small cohort of normal patients from a previous study.^42^

The high prevalence of abnormal anterior topography points to a genetic basis for KC in Israel. The expectation would be that in population in which KC is a dominant monogenic disease, a large percent of family members will have manifest KC. Alternatively, in a population in which it is a complex disorder involving several genes and environmental triggers, we would expect to see an increased prevalence of various corneal abnormalities in family members, although not necessarily manifest disease. The results of the study support the later scenario: KC in Israel appears to be a complex disorder. The first‐degree relatives have a larger number of corneal abnormalities than controls. This implies that KC is caused by changes at many genes and each will contribute to the shape of the cornea: some will impact corneal thickness, others anterior curvature and perhaps even others will be responsible for posterior curvature. Furthermore, there was no evidence of sex‐linkage. Male and female relatives had a similar prevalence of abnormalities.

*Table* *6* compares the results of this study to previous investigations of the corneal phenotype of KC family members using topography or tomography. The methodologies that were used in each paper differ from the current study and from one another. Some determined the prevalence of KC or KC suspect,^34^, ^37^, ^38^, ^39^, ^40^ while others looked at various corneal abnormalities as determined by corneal topography and/or tomography anterior and posterior curvature,^35^, ^36^, ^41^ and still others look at higher order aberrations.^49^, ^50^, ^51^, ^52^ The definition of abnormalities was not the same in the various studies since they used different tomography/topography instruments each with its unique parameters. Only Kaya *et al*.,^39^ and Awwad *et al*.,^38^ had the same study group as the current research: first‐degree relatives in families in which KC was sporadic (only one member identified as having KC at the onset of the research.) However, they limited their analysis to the prevalence of manifest KC. The current study added to these findings by providing a thorough phenotyping of the corneas of first‐degree relatives. Furthermore, it is the first study of its kind carried out in Israel, a country that has been shown to have a high prevalence of KC.^13^, ^14^

**Table 6:** Prevalence of keratoconus and corneal abnormalities in family members of KC probands

Study	Location	Participants	Family relations	% KC	% KC suspect	% abnormal corneas
						Topographic abnormality
Studies with topography						
Rabinowitz *et al.* ^36^ 1990	USA	KC: 5, Family: 28	Extended – familial^†^	N/A	N/A	50%
Wang *et al.* ^37^ 2000	USA	KC: 381, Family: 373	First‐degree, sporadic^‡^	3.3%	N/A	N/A
Levy *et al.* ^35^ 2004	France, Spain	KC: 55, Family: 132	Extended, familial	N/A	N/A	25.8%
Karimian *et al.* ^34^ 2008	Iran	KC: 45, Family: 150	Extended, sporadic	12.3%	6.7%	N/A
Studies with tomography					
Kaya *et al.* ^39^ 2007	Turkey	KC: N/A, Family: 72	First‐degree, sporadic	11%	N/A	N/A
Steele *et al.* ^41^ 2008	Australia	KC: 11, Family: 90	Extended, familial	N/A	N/A	25%
Kymionis *et al.* ^40^ 2017	Greece	KC: 51; Family: 34	Extended – familial	23%	N/A	53%
Awwad *et al.* ^38^ 2019	Lebanon	KC: 124, Family: 183	Pediatric First‐degree, sporadic	17‐19%	N/A	N/A
Current study	Israel	KC: 16, Family: 56	First ‐degree, sporadic	4%	14%	34%

Abbreviations: Extended, first and second‐degree relatives were included in the study; Familial, families known to have several members with KC; First‐degree, only first‐degree relatives were included in the study; KC, keratoconus; sporadic, families with only one known case of KC, N/A, not applicable.

^†^ Families with several keratoconus patients.

^‡^ Keratoconus subjects from families with sporadic disease.

In Turkey,^39^ Lebanon,^38^ Iran^34^ and Greece,^40^ the prevalence of manifest KC in KC relatives is much higher than in Israel (*Table* *6*). In contrast, the prevalence in Israel and the United States^37^ are similar, as is the prevalence of corneal abnormalities between Israel and Australia.^41^ This suggests two different patterns of inheritance. Perhaps in Turkey, Lebanon, Iran and Greece there is a dominant mechanism with partial penetrance, while in Israel, the US and Australia, KC a complex disorder with several genes and environmental triggers. Alternatively, the higher prevalence in Greece may be due to the inclusion of families with a history of KC.

The qualitative patterns of anterior corneal topography found in this study was similar to that in previous studies of KC relatives and controls.^35^, ^40^ A large percent of the KC relatives had abnormal patterns especially a skewed bowtie with inferior steepening, while this pattern is rare in controls. Conversely, controls have a much higher prevalence of normal patterns (oval, symmetric bowtie), than KC relatives. This highlight the importance of testing first‐degree family members. While the procedure used in this study, to evaluate anterior topography might be cumbersome for clinicians, it provides an easy to use tool that does not depend on the more expensive corneal tomography instruments.

Age appears to be a risk factor for corneal abnormalities for the first‐degree relatives but not for control subjects. This suggests that some of the KC first‐degree relatives may develop KC in the future. Clinicians should recommend longitudinal observation of relatives of KC patients, especially if they are young.

### Limitations

Despite having a large patient base of KC patients at the HAC clinic, only 16 KC patients complied with the recommendation to bring in first‐degree relatives for a complete exam. The low compliance may have caused an ascertainment bias in which families who suspected that they had KC were more likely to come for full exams. Another limitation is percent of women was much larger in the control group than in the first‐degree relatives. Since we did not find a sex linkage with corneal abnormalities this is not likely to have impacted the results.

Another limitation of this study was that the sample size was too small to have an 80% power in determining if corneal abnormalities were linked to sex and further research must be carried out on this topic.

Lastly, the clinicians were not masked to the identity of the patients and their images (first‐degree relatives or controls) and this may have added bias to the study.

## Conclusions

In Israel, KC does not appear to follow a simple Mendelian pattern of inheritance. Clinicians should consider first‐degree relatives of patients with KC at moderate risk for the disease and/or corneal abnormalities, especially in younger patients. A full clinical exam including corneal topography assessment it is recommended in this cohort of patients with frequent follow‐up.

## Conflict of interest

The authors report no conflicts of interest and have no proprietary interest in any of the materials mentioned in this article.

## Supporting information


**Table S1.** (a) Anderson‐Darling Normality Test results and median, 25th and 75th percentile for each parameter. If normality tests of both first‐degree relatives and Healthy controls were above 0.05, then T‐test was performed, otherwise, Mann Whitney U test. Abbreviations: K, keratometry reading; CCT, central corneal thickness; TCT, Thinnest corneal thickness; SIf, symmetry index front; SIb, symmetry index back; BCVf, Baiocchi Calossi Versaci front; BCVb, Baiocchi Calossi Versaci back; RMSf, root mean square front; RMSb, root mean square back. (b) Skewness Normality Test results. A result between ± 1.6 is considered normally distributed.

**Table S2.** Quantitative analysis of corneal abnormalities. Any elevation defect represents a defect at least one of the following parameters: BCVf, BCVb, RMSf and RMSb. Any defect represents indicates subjects with at least one of the other abnormalities in the figure. Abbreviations: KC, keratoconus; TCT, Thinnest corneal thickness; SIf, symmetry index front; SIb, symmetry index back; BCVf, Baiocchi Calossi Versaci front; BCVb, Baiocchi Calossi Versaci back; RMSf, root mean square front; RMSb, root mean square back; ^†^, Chi‐square; ^‡^, Fischer’s exact test.

**Table S3.** Prevalence of topography patterns in family members and normal controls. *p* value is between family members and healthy controls. Abbreviations: SB, symmetric bowtie; AB, asymmetric bowtie; SRAX, with skewed radial axes; IS, inferior steepening; SS, superior steepening; χ^2^, Chi‐square test.
