# Supplementary material for: The prevalence of corneal abnormalities in first‐degree relatives of patients with keratoconus: a prospective case‐control study
Source: Ophthalmic Physiol Opt. 2020 Jul 24;40(4):442–51. doi: 10.1111/opo.12706 (PMC7496242; doi:10.1111/opo.12706)
Supplement: Supplementary file 1 — Table S1. (a) Anderson‐Darling Normality Test results and median, 25th and 75th percentile for each parameter. If normality tests of both first‐degree relatives and Healthy controls were above 0.05, then T‐test was performed, otherwise, Mann Whitney U test. Abbreviations: K, keratometry reading; CCT, central corneal thickness; TCT, Thinnest corneal thickness; SIf, symmetry index front; SIb, symmetry index back; BCVf, Baiocchi Calossi Versaci front; BCVb, Baiocchi Calossi Versaci back; RMSf, root mean square front; RMSb, root mean square back. (b) Skewness Normality Test results. A result between ± 1.6 is considered normally distributed. [file OPO-40-442-s001.pdf]

Table 1A supplementary data. Anderson-Darling Normality Test results and median, 25th and 75th percentile for each parameter. If normality tests of both First-degree relatives and Healthy controls were above 0.05, then T-test was performed, otherwise, Mann Whitney U test. Abbreviations: K, keratometry reading; CCT, central corneal thickness; TCT, Thinnest corneal thickness; SIf, symmetry index front; SIb, symmetry index back; BCVf, Baiocchi Calossi Versaci front; BCVb, Baiocchi Calossi Versaci back; RMSf, root mean square front; RMSb, root mean square back.

	**First-degree relatives (25th, median and 75th percentile)**	**Healthy controls (25th, median and 75th percentile)**
**Anterior K1**	0.03<br>(7.6, 7.8, 7.9)	0.13<br>(7.6, 7.7, 7.9)
**Anterior K2**	0.01<br>(7.4, 7.6, 7.8)	0.10<br>(7.4, 7.6, 7.7)
**Average Anterior K**	0.01<br>(7.5, 7.7, 7.8)	0.07<br>(7.5, 7.7, 7.8)
**Posterior K1**	0.03<br>(6.4, 6.7, 6.8)	0.18<br>(6.4, 6.6, 6.7)
**Posterior K2**	<0.0001<br>(6.1, 6.3, 6.5)	0.19<br>(6.0, 6.2, 6.4)
**Average Posterior K**	0.001<br>(6.3, 6.5, 6.6)	0.07<br>(6.2, 6.4, 6.6)
**Front Apex Thickness**	0.65<br>(522.7, 553.5, 599.3)	0.001<br>(541.2, 568.2, 626.2)
**Front Apex Curve**	<0.0001<br>(7.2, 7.4, 7.7)	0.33<br>(7.2, 7.4, 7.6)
**Back Apex Curve**	<0.0001<br>(5.6, 5.8, 6.1)	0.33<br>(5.6, 5.8, 6.0)

**CCT**	0.54<br>(508.1, 534.4, 563.2)	0.38<br>(523.1, 544.4, 562.5)
**TCT**	0.20<br>(506.0, 529.9, 560.6)	0.36<br>(518.4, 540.8, 560.4)
**SIf**	<0.0001<br>(-0.1, 0.2, 0.5)	0.08<br>(-0.1, 0.1, 0.5)
**SIb**	<0.0001<br>(0.0, 0.1, 0.1)	0.26<br>(-0.1, 0.0, 0.1)
**BCVf**	<0.0001<br>(0.0, 0.2, 0.4)	<0.0001<br>(0.0, 0.1, 0.3)
**BCVb**	<0.0001<br>(0.0, 0.0, 0.2)	<0.0001<br>(0.0, 0.0, 0.1)
**RMSf**	<0.0001<br>(2.0, 2.6, 4.0)	<0.0001<br>(1.8, 2.2, 2.9)
**RMSb**	<0.0001<br>(6.1, 7.5, 9.1)	0.001<br>(4.7, 5.8, 7.5)

Table 1B supplementary data. Skewness Normality Test results. A result between ±1.6 is considered normally distributed.

	**First-degree relatives**	**Healthy controls**
**Sphere**	-1.43	-1.04
**Cylinder**	-1.37	-1.67
**Anterior K1**	0.86	-0.84
**Anterior K2**	0.93	-1.09
**Average Anterior K**	0.94	-0.98
**Posterior K1**	0.49	-0.78
**Posterior K2**	0.47	-1.62
**Average Posterior**	0.53	-1.26
**Front Apex Thickness**	0.56	0.06

**Front Apex Curve**	-0.03	-2.15
**Back Apex Curve**	-0.03	-2.11
**CCT**	0.58	-0.49
**TCT**	0.59	-0.77
**SIf**	-0.56	4.94
**SIb**	-0.42	5.72
**BCVf**	1.07	4.18
**BCVb**	2.28	5.87
**RMSf**	1.17	2.86
**RMSb**	1.30	3.14
