# Supplementary material for: The prevalence of corneal abnormalities in first‐degree relatives of patients with keratoconus: a prospective case‐control study
Source: Ophthalmic Physiol Opt. 2020 Jul 24;40(4):442–51. doi: 10.1111/opo.12706 (PMC7496242; doi:10.1111/opo.12706)
Supplement: Supplementary file 2 — Table S2. Quantitative analysis of corneal abnormalities. Any elevation defect represents a defect at least one of the following parameters: BCVf, BCVb, RMSf and RMSb. Any defect represents indicates subjects with at least one of the other abnormalities in the figure. Abbreviations: KC, keratoconus; TCT, Thinnest corneal thickness; SIf, symmetry index front; SIb, symmetry index back; BCVf, Baiocchi Calossi Versaci front; BCVb, Baiocchi Calossi Versaci back; RMSf, root mean square front; RMSb, root mean square back; †, Chi‐square; ‡, Fischer’s exact test. [file OPO-40-442-s002.pdf]

Table 2 Supporting information. Quantitative analysis of corneal abnormalities. Any elevation defect represents a defect at least one of the following parameters: BCVf, BCVb, RMSf and RMSb. Any defect represents indicates subjects with at least one of the other abnormalities in the figure. Abbreviations: KC, keratoconus; TCT, Thinnest corneal thickness; SIf, symmetry index front; SIb, symmetry index back; BCVf, Baiocchi Calossi Versaci front; BCVb, Baiocchi Calossi Versaci back; RMSf, root mean square front; RMSb, root mean square back; †, Chi-square; ‡, Fischer's exact test.

**Parameter**	**KC first-degree relatives % abnormal (N=56)**	**Healthy Controls % abnormal (N=96)**	**Chi square P value* (1, N = 152)**
**TCT<482.92**	13%	1%	P = 0.01‡
**SIf >0.7774**	21%	11%	χ^2= 2.74 P = 0.1†
**SIb >0.2395**	13%	0%	P = 0.001‡
**Any curvature defect (Sif or SIb)**	25%	11%	χ^2= 4.72 P = 0.03†
**BCVf >0.5858**	14%	3%	P = 0.05‡
**BCVb >0.6772**	7%	0%	P = 0.05‡
**RMSf >7.2975**	14%	0%	P < 0.0001‡
**RMSb >13.9459**	13%	1%	P = 0.01‡
**Any elevation defect (BCVf or BCVb Or RMSf OR RMSb)**	18%	3%	P = 0.01‡
**Thickness, curvature or elevation defect**	34%	14%	χ^2= 8.84 P = 0.01†
