# Supplementary material for: The prevalence of corneal abnormalities in first‐degree relatives of patients with keratoconus: a prospective case‐control study
Source: Ophthalmic Physiol Opt. 2020 Jul 24;40(4):442–51. doi: 10.1111/opo.12706 (PMC7496242; doi:10.1111/opo.12706)
Supplement: Supplementary file 3 — Table S3. Prevalence of topography patterns in family members and normal controls. p value is between family members and healthy controls. Abbreviations: SB, symmetric bowtie; AB, asymmetric bowtie; SRAX, with skewed radial axes; IS, inferior steepening; SS, superior steepening; χ2, Chi‐square test. [file OPO-40-442-s003.pdf]

Table 3 Supporting information. Prevalence of topography patterns in family members and normal controls. P value is between family members and healthy controls. Abbreviations: SB, symmetric bowtie; AB, asymmetric bowtie; SRAX, with skewed radial axes; IS, inferior steepening; SS, superior steepening; χ^2, Chi-square test.

	KC first-degree relatives	Healthy control	
**AB SRAX IS**	11%	5%	
**AB SRAX SS**	4%	1%	
**AB SRAX**	2%	0	
**IS**	16%	8%	
**Irregular**	2%	2%	
**AB SS**	5%	7%	
**AB IS**	16%	14%	
**Oval**	7%	20%	
**Round**	13%	25%	
**SB**	23%	14%	
**SS**	2%	4%	
**Abnormal Topography**	34% (19,37)	17% (16,80)	$\chi^2_{(1, N=152)} = 5.95$ P = 0.02
